# Study on Bulk Texture and Mechanical Properties of As-Extruded Wide Mg-Al-Zn Alloy Sheets with Different Al Addition

**DOI:** 10.3390/ma15124147

**Published:** 2022-06-10

**Authors:** Yu-Qing Li, Da-Ye Xu, Min Zha, Dong-Feng Chen, Yun-Tao Liu, Mei-Juan Li, Kai Sun, Gui-Jie Zhu, Si-Qing Wang, Tong Wang, Jian-Bo Gao, Xiao-Long Liu

**Affiliations:** 1 China Institute of Atomic Energy, Beijing 102413, China; liyuqing@ciae.ac.cn (Y.-Q.L.); dongfeng@ciae.ac.cn (D.-F.C.); zhuguijie@ciae.ac.cn (G.-J.Z.); liuxiaolong@ciae.ac.cn (X.-L.L.); 2 Department of Physics, Renmin University of China, Beijing 100872, China; xudaye@ruc.edu.cn; 3 Key Laboratory of Automobile Materials of Ministry of Education & School of Materials Science and Engineering, Jilin University, Changchun 130025, China; minzha@jlu.edu.cn (M.Z.); wangsq20@mails.jlu.edu.cn (S.-Q.W.); wangt19@mails.jlu.edu.cn (T.W.); 4 Centre of Excellence for Advanced Materials, Dongguan 523808, China; jianbo.gao@ceamat.com

**Keywords:** wide magnesium alloy sheets, extrusion, microstructure, bulk texture, mechanical properties

## Abstract

The wide Mg alloy sheets produced by hot extrusion usually can easily form an inhomogeneous texture, resulting in anisotropic mechanical properties and poor formability. However, few studies have been carried out on the bulk texture investigation at different areas of as-extruded Mg alloy sheets, especially the Mg alloys with different alloying elements. In this work, the effect of Al on the bulk texture and mechanical properties at different areas for three wide Mg-Al-Zn alloy sheets with different Al contents (Mg-3Al-0.5Zn, Mg-8Al-0.5Zn and Mg-9Al-0.5Zn) are mainly investigated by neutron diffraction. The results showed that a strong and uneven basal texture was formed in the Mg-3Al-0.5Zn sheet. Meanwhile, the intensity of the basal texture was significantly weakened due to the numerous fine precipitates of Mg_17_Al_12_ particles, with the Al content increasing, which hinder the grain growth during extrusion, while fine recrystallized grains have a more random orientation. The enhanced tensile properties in Mg-8Al-0.5Zn and Mg-9Al-0.5Zn alloy sheets are ascribed to the cooperation effect of a refined microstructure, precipitates and weakened basal texture. Among the three Mg alloy sheets, the Mg-8Al-0.5Zn alloy sheet has a yield strength of about 270 MPa, an ultimate tensile strength of about 330 MPa and ultimate elongation of about 16% in the extrusion direction, which possesses the most excellent comprehensive mechanical properties.

## 1. Introduction

Facing the urgent demands in environmental protection and energy saving, magnesium (Mg) alloys, as the lightest metallic structure material, attract researchers’ attention [1,2,3]. In recent years, Mg alloys have been of great potential for applications in the fields of aerospace, automobiles and electronics due to their high specific strength, stiffness and so on [4,5,6,7]. However, Mg alloys usually possess poor formability and limited ductility at room temperature, which severely restricts the application of wrought Mg alloys. Numerous attempts have been made to get an excellent balance of strength and ductility of commercial wrought Mg alloys. Grain refinement and precipitation hardening, especially texture modification, have been proved to be effective methods for enhancing the mechanical properties of wrought Mg alloys [8,9,10,11,12,13].

According to the previous research, the formability of Mg alloy sheets was strongly affected by the initial crystallographic texture of the (0002) basal plane. Usually, Mg alloy sheets easily produce a strong basal texture during the primary process, such as hot rolling, extrusion or other deformation processing. This can result in limited room temperature formability and strong anisotropy [14,15,16]. Thus, texture control during the primary process should be considered as a very effective way to enhance formability in the following process. Mg alloys always need a combination of deformation modes because of a lack of independent deformation systems. On the one hand, this makes the deformation more complicated during the deformation process. On the other hand, the influence of texture is more evident in the material’s properties. An extrusion process aimed to modify the texture of Mg alloy sheets, including the tilted orientation, the inclined degree of the basal plane and the texture intensity has been developed to modify the basal texture of Mg alloy sheets [17,18]. Therefore, this process is an industrially available and effective method to control texture and improve the mechanical properties of Mg alloys.

To optimize mechanical properties, many investigations have been carried out on various novel extruded Mg alloys, as alloying elements influence the microstructure, texture and properties greatly. It has been proved that the addition of rare-earth (RE) elements into Mg alloys can significantly weaken the basal texture, activate non-basal slip systems and refine grain size. However, RE elements are rare and high-cost [17,18,19]. Hence the RE-free Mg alloys are much more competitive for commercial utilizations. The Zn element is a commonly used alloying element in Mg alloys, which can refine the grain size and contribute to solid solution and aging strengthening simultaneously [20,21]. Additionally, an Al addition can contribute to the decrease in the stacking fault energies in Mg alloys, and hence be beneficial to the strong strain hardening ability and high elongation [22]. Mg-Al-Zn (AZ) system alloys have received progressive interest due to their low cost, good precipitation hardenability and creep resistance among the RE-free Mg alloys.

At present, many studies have been carried out on the microstructure, texture and properties of AZ system alloys. Wang et al. carried out a quantitative analysis of the microstructure and mechanical properties of hot-extruded AZ31 Mg alloys with different grain sizes. They found deformation modes of grains were dependent on the orientation of grains [14]. Xu et al. found that the AZ91 alloy prepared by equal channel angular pressing (ECAP) exhibited an anisotropic mechanical behavior, which can be well explained by texture [23]. Gryguc et al. reported that a sharp basal texture in extruded AZ80 resulted in tension–compression asymmetry in both the monotonic and cyclic responses [24]. Zhang et al. studied AZ31, AZ61 and AZ91 alloys prepared by novel hard-plate rolling (HPR). The results showed the well-maintained fine equiaxed grain structures accompanied by the greatly weakened basal texture in the AZ91 alloy, suggesting that grain boundary sliding (GBS) is the dominant superplastic deformation mechanism [25]. However, up to now, systematic investigations on the effect of the Al content on the microstructure and bulk texture evolution of extruded wide Mg alloy plates are still limited, especially utilizing neutron diffraction to obtain reliable texture measurements. For this reason, in this study, Mg-xAl-0.5Zn-0.2Mn (wt.%) alloy wide thick plates with different contents of Al were chosen to reveal the effects of Al additions on the microstructure, texture and mechanical properties of as-extruded AZ wide sheets.

Many papers have been published to describe different kinds of influences on the texture development of as-extruded AZ Mg alloys [14,26,27,28,29,30,31,32]. In previous studies, the texture of as-extruded Mg alloy sheets is mainly characterized by EBSD and X-ray diffraction (XRD). Usually, these techniques are used to measure the local and surface textures in materials. However, for industry and practical engineering applications, the bulk texture of large-scaled materials is more important than the local texture [33,34]. Moreover, coarse grains may be produced in Mg alloy sheets during extrusion. Texture measurements by EBSD and XRD lack representation due to poor statistics.

Neutrons only interact with nuclei, so they have a strong ability of penetration in materials [35,36,37], which means that neutron diffraction has the following advantages in texture measurement among these techniques. For example: (1) large samples can be used, thus a high accuracy and good statistics, as well as bulk texture, will be obtained; (2) accurate texture analysis can be achieved for coarse-grained, texture inhomogeneity, multi-phase samples and samples with a small volume fraction of the second phase; (3) texture evolution during a high temperature and tension process can be studied by in situ neutron diffraction. At present, neutron diffraction has become a standard method to investigate bulk textures of different types of materials. Table 1 presents the typical penetration depth and sample volume of three kinds of radiation [38]. However, the neutron diffraction method has its limitations. For example, the neutron texture diffractometer is complex, expensive and relatively difficult to operate and maintain, hence a neutron beam is expensive and not easy to get. Meanwhile, data analysis is relatively difficult due to different data acquisitions and analysis systems for each neutron texture diffractometer. Therefore, there are few reports on the study of texture evolution of Mg alloys by neutron diffraction.

Normally, the different stresses imparted at the edge and center areas may cause an inhomogeneous texture and performance in wide as-extruded Mg alloy sheets. However, few studies have been carried out on the texture investigation at different areas of extruded Mg alloy sheets, especially the bulk texture study using neutron diffraction. In this study, the texture variations at different areas of the three as-extruded Mg-Al-Zn alloy sheets with different Al contents are characterized by neutron diffraction, as well as the tensile properties being tested. This work aims to reveal the influence of the Al content on the bulk texture and, further, on mechanical properties, providing guidance for texture control via the tailoring alloying composition and development of Mg alloy sheets suitable for extrusion processing.

## 2. Materials and Methods

In this work, Mg-Al-Zn-based alloys with different contents of Al were prepared from commercial pure Mg (99.85 wt.%), pure Al (99.90 wt.%) and pure Zn (99.90 wt.%). The alloying components were completely melted in an electric resistance furnace under the protection of a gas mixture of CO_2_ and SF_6_. Then the melt was poured into a steel mold. The cast ingots were subsequently homogenized at 430 °C for 3 h in a resistance furnace. The hot extrusion was then performed with a mold temperature of ~420–430 °C and extrusion ratio of ~29 for AZ31, and an initial mold temperature of ~360–370 °C for AZ81 and AZ91. The extrusion ratio for AZ81 and AZ91 are about ~17 and ~29, respectively. Finally, the as-extruded AZ81 alloy sheet had a final cross section of about 330 mm (width) × 14 mm (thickness), and AZ31 and AZ91 alloy sheets had a final cross section of about 350 mm (width) × 8 mm (thickness). The measured chemical compositions of the studied alloys were listed in Table 2.

The microstructure of as-extruded alloys was examined by optical microscopy (OM: MV3000), field emission scanning electron microscopy (FESEM: ZEISS ULTRA 55 and FEI QUANTA FEG 450) equipped with an energy dispersive spectrometer (EDS) analyzer (INCA X-MAX 50 and TEAM EDS) and Field emission electron backscattering diffraction (EBSD: ZEISS ULTRA 55) equipped with analyzer (HKL Channel 5) in ED-TD plane (ED: extrusion direction, TD: transverse direction). The metallographic samples for OM, FESEM and EBSD microstructure observations were prepared by conventional grinding and electro polishing at a voltage of 20 kV with a current of 0.4 A for 60 s at room temperature. The samples for OM were also chemically etched in acetic picric solution (100 mL ethanol, 5 g picric acid, 5ml acetic acid, 10 mL distilled water) for 10 s. Phase analyses were performed by a Bruker D8 Advanced diffractometer with a wavelength of 1.5406 Å (Cu-K_α_ radiation) and the scanning step of 0.02° between 10° and 90° (2theta).

**Figure 1:**
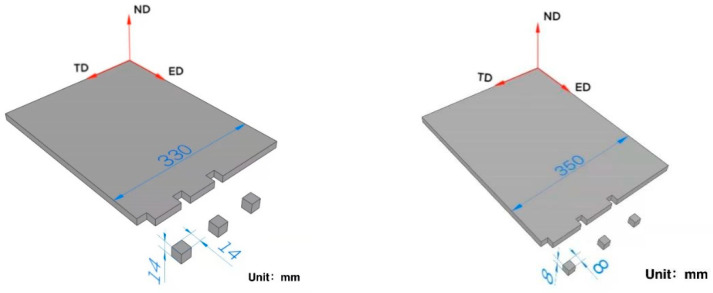
Sketch of sample preparation for texture measurement.

Neutron diffraction was used to investigate the bulk texture distribution in Mg alloy sheets in this study. To get texture variation in different positions, samples were taken from edge, 1/4 and center positions along TD of alloy sheets, respectively. Cubic samples with a size of 8 mm × 8 mm × 8 mm from AZ31 and AZ91 alloys, 14 mm × 14 mm × 14 mm from AZ81 alloy, which are suitable for neutron texture measurement, were cut by wire-electrode cutting. Figure 1 shows the sketch of samples’ preparation for texture measurement, correspondingly, the number #1, #2 and #3 represent samples from positions of edge, 1/4 and center. Then the (10–10), (0002) and (10–11) complete pole figures were measured using the neutron texture diffractometer at China Advanced Research Reactor. The texture of Mg alloy sheets was measured at a neutron beam wavelength of 1.48 Å. The tested samples were mounted on a Eulerian cradle and their corresponding diffraction images were recorded using a position sensitive area detector. For measurement of the complete pole figure, samples were both tilted (χ = 0–90°) with an angle interval of 15° and rotated (ϕ = 0–360°) with an angle interval of 5°. The measurement time of each data point was 10 s. The software of LaboTex was used to analyze the data and plot the pole figures.

Tensile tests were carried out using CMT5605 universal tensile testing machine at room temperature under a constant strain rate of about 0.7 × 10^−3^ s^−1^. Tensile specimens were cut from as-extruded sheets with the tensile axis parallel to the ED, and the gage size is 50 × 12.5 × 3.5 mm^3^. At least 3 samples were tested and stress–strain curves with good repeatability are presented.

## 3. Results and Discussion

### 3.1. Microstructural Characteristics

**Figure 2:**
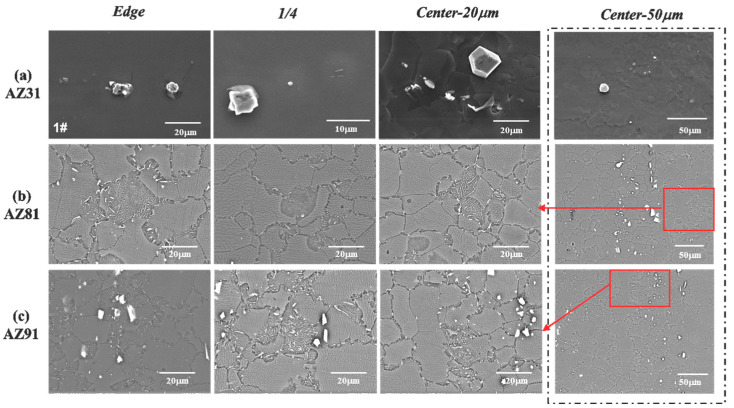
SEM pictures of three as-extruded Mg alloy sheets in different positions: (**a**) AZ31; (**b**) AZ81; (**c**) AZ91.

The microstructure of three alloys was examined using SEM and the results are presented in Figure 2. It can be seen that there are a few coarse flake precipitates distributed unevenly in the AZ31 alloy sheet. These precipitates are in a square, polygonal or strip shape with different sizes in diagonal length, and the biggest is nearly 20 μm. However, the precipitates increase with the increase of the Al content, while the size of precipitated particles decreases. In AZ81 and AZ91 alloy sheets, a large number of precipitates, which include fine strip particles mainly distributed along the grain boundary, are formed. Meanwhile, some coarse flaky particles with an irregular shape are distributed along the grain boundary or in the grain. It is noteworthy that there are some discontinuous regions in which a large number of long filamentous precipitates exist in the AZ81 and AZ91 alloy sheets. They are very similar to the morphology of cellular discontinuous precipitates in Mg alloys reported in the previous literature [39].

**Figure 3:**
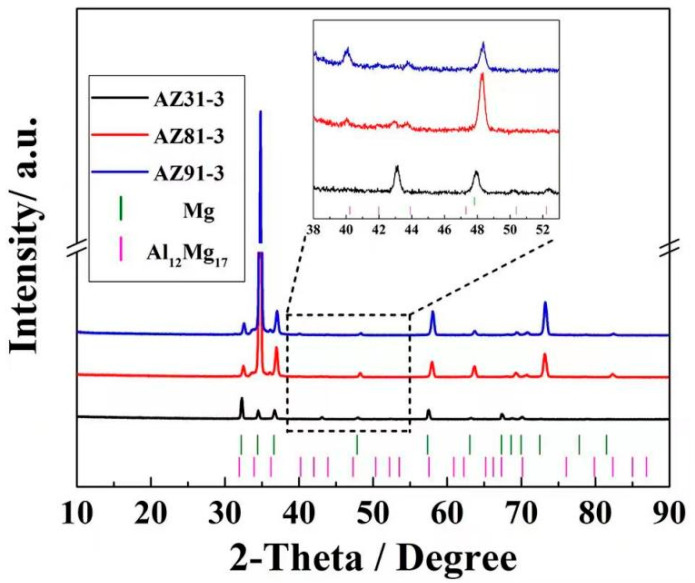
X-ray diffraction spectra of three as-extruded Mg alloy sheets in center area.

X-ray diffraction (XRD) analyses are shown in Figure 3 for three Mg alloy sheets in center position. No Mg_17_Al_12_ (PDF#01-1128) particles are found in AZ31 sheet except the Mg matrix phase (PDF#35-0821), but some weak diffraction peaks of other components exist in the AZ31 sheet, indicating that there may be a small amount of other second phase particles in AZ31, which is consistent with the SEM results in Figure 2a. However, due to the small content, the diffraction peaks cannot be well fitted and analyzed. With the increase of Al content, some weak diffraction peaks of Mg_17_Al_12_ can be observed at low angles of 2theta in the AZ81 and AZ91 sheets, and the peaks seem stronger in AZ91 than in the AZ81 sheet. However, the diffraction peaks of Mg_17_Al_12_ are hardly visible at the high degree of 2theta, which may attribute to the poor resolution of XRD in high angles and a small content of Mg_17_Al_12_. It is worth noting that α-Mg diffraction peaks shift towards high 2theta angles in all three Mg alloys. Moreover, with an increase in the Al content in Mg alloys, the diffraction peaks shift more toward high angles. Considering that the atom radius of Al and Zn are all smaller than Mg, we may conclude that more solute atoms have been dissolved into the Mg matrix with the increase in the Al content, resulting in an interplanar spacing decrease. According to the Bragg equation 2dsinθ = λ, when the X-ray wavelength λ is fixed, the smaller interplanar spacing d is, the larger diffraction 2theta is. Therefore, with the increase in the Al content in the Mg alloys, the diffraction peak gradually shifts to a high 2theta angle.

**Figure 4:**
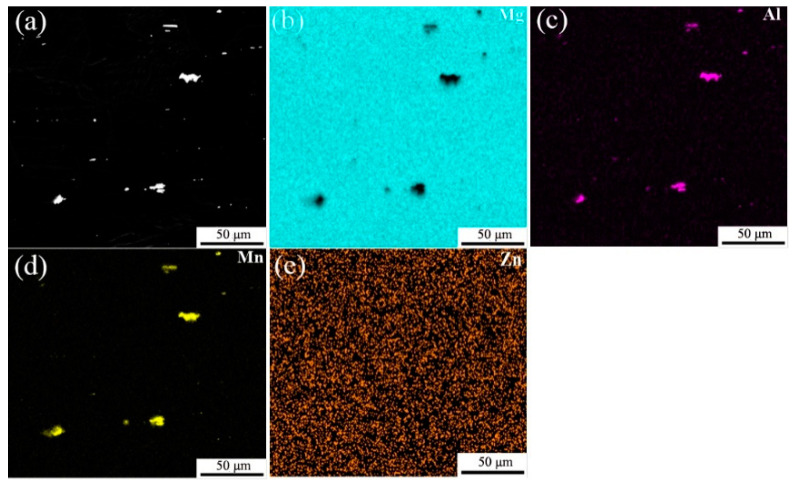
EDS mapping of the main alloying elements of as-extruded AZ31 in center area: (**a**) SEM image; (**b**) Distribution of Mg element; (**c**) Distribution of Al element; (**d**) Distribution of Mn element; (**e**) Distribution of Zn element.

**Figure 5:**
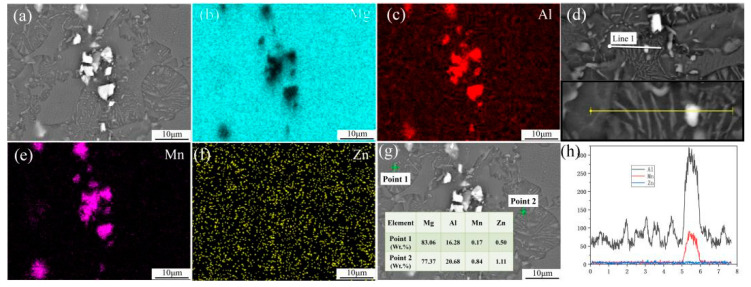
EDS mapping of the main alloying elements, spot and linear analysis of as-extruded AZ91 in center area: (**a**) SEM image; (**b**) Distribution of Mg element; (**c**) Distribution of Al element; (**d**) Linear scanning; (**e**) Distribution of Mn element; (**f**) Distribution of Zn element; (**g**) Main element content obtained by spot analysis; (**h**) Element spectrum of Al, Mn and Zn obtained by linear scanning.

EDS analysis of as-extruded AZ31 and AZ91 alloy sheets in center areas were conducted and the results are presented in Figure 4 and Figure 5, respectively. EDS mapping of the main alloying elements indicates that the coarse flake precipitates in AZ31 mainly consist of a high content of Al and Mn elements, while the Zn element distributes uniformly both in the a-Mg matrix and precipitates (Figure 4). Actually, it has been reported that the Zn present in the AZ91 alloy does not create new phases. Thus one can infer that the coarse flaky particles in the AZ31 alloy sheet are Al_2_Mn particles. The EDS mapping for the AZ91 alloy sheet reveals that the coarse flake precipitates are also Al_2_Mn. The spot analysis was carried out on filamentous precipitates in cellular discontinuous regions, and the result shows that a high content of Mg and Al elements are detected in filamentous precipitates, which indicates that Mg_17_Al_12_ particles are precipitated in the AZ91 sheet. The EDS line analysis also confirms the coarse flaky particles are Al_2_Mn and filamentous particles are Mg_17_Al_12_. It can be seen that when the Al content is relatively low, for example, AZ31, only the Al_2_Mn precipitates are formed. With the increase in the Al content, Mg_17_Al_12_ particles are formed by the dynamic precipitation of supersaturated solid solution during hot extrusion.

**Figure 6:**
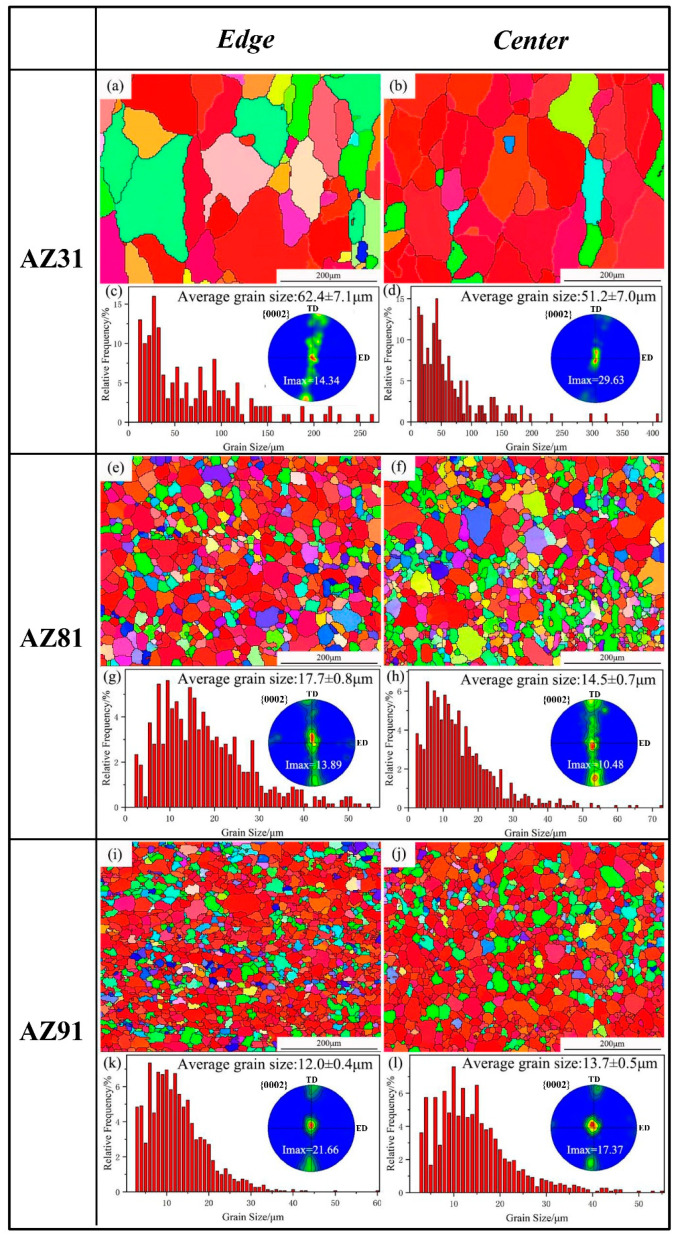
EBSD orientation maps, (0002) pole figure, grain size distribution and average grain size in edge and center of as-extruded three Mg alloy sheets: (**a**,**c**) The edge of AZ31; (**b**,**d**) The center of AZ31; (**e**,**g**) The edge of AZ81; (**f**,**h**) The center of AZ81; (**i**,**k**) The edge of AZ91; (**j**,**l**) The center of AZ91.

EBSD characterization of the sheets was performed on the edges and central areas of as-extruded Mg alloy sheets with different components. Figure 6 shows the inverse pole figure, texture and grain size distribution of three Mg alloy samples. As shown in Figure 6a,b, the AZ31 alloy sheet exhibits a typical bimodal microstructure which consists of a large number of coarse grains and a small number of fine grains. The average grain size, with the error bars corresponding to the 95% confidence limits of the edge and center parts, are 62.4 μm and 51.2 μm, respectively. As illustrated in Figure 6e,f,i,j, the grains are greatly refined compared to the AZ31 alloy sheet, although there are still a few coarse grains in the AZ81 and AZ91 sheets. The AZ91 sheet possesses the most uniform structure and finest grains among all three alloy sheets, and the average grain sizes in the edge and center positions are 12.0 μm and 13.7 μm, respectively, which are much smaller in comparison to the AZ31 alloy sheet. According to the study [27], Mg_17_Al_12_ particles at the grain boundaries effectively hinder the growth of dynamic recrystallized (DRXed) α-Mg grains, which leads to the stabilization of DRXed grain size during high-temperature extrusion. It can be seen from Figure 2, Figure 3, Figure 4 and Figure 5 that a large number of Mg_17_Al_12_ particles precipitate at the grain boundaries in AZ81 and AZ91, however, only coarse Al_2_Mn particles were formed in AZ31. Therefore, AZ81 and AZ91 alloy sheets have a much smaller grain size compared to the AZ31 sheet due to the existence of Mg_17_Al_12_ particles distributed at grain boundaries. On the other hand, the hot extrusion temperature for AZ31 (with a mold temperature of ~420–430 °C) is higher than that for AZ81 and AZ91 (with a mold temperature of ~360–370 °C), meaning the grains may grow up quickly in the AZ31 sheet after complete dynamic recrystallization. In total, the differences in the precipitation and extrusion temperature are the two main reasons which cause the formation of coarse grains in the AZ31 sheet.

### 3.2. Bulk Texture

**Figure 7:**
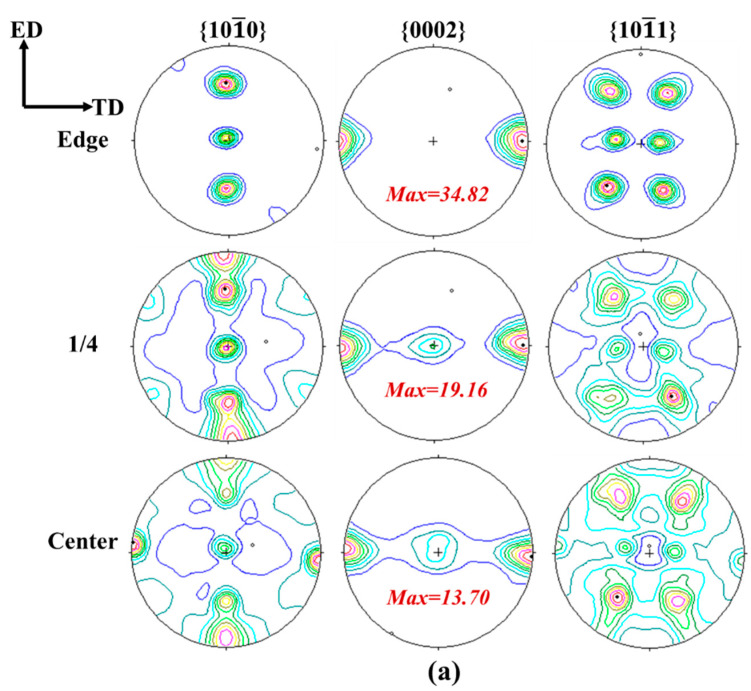

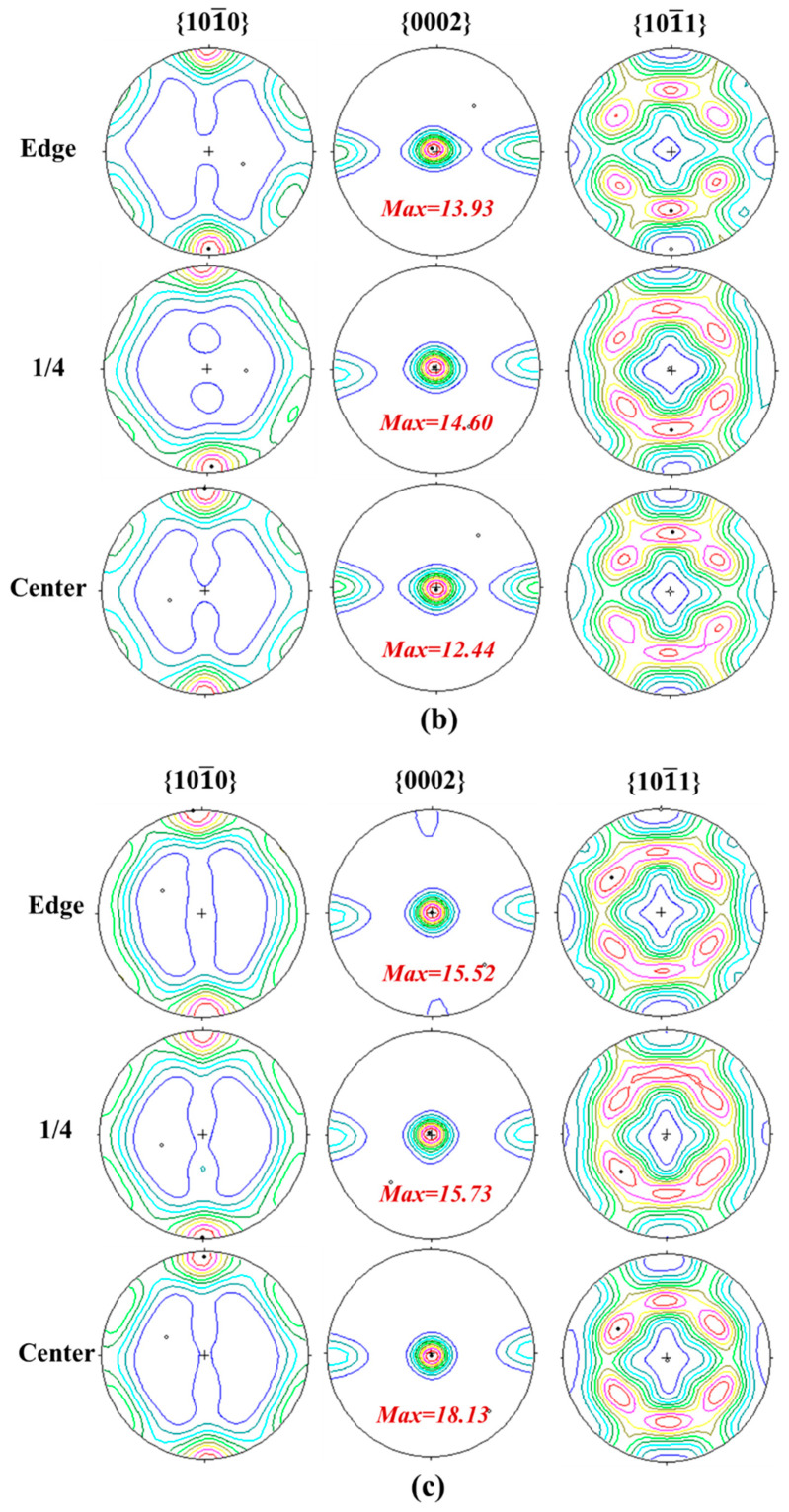
(10–10), (0002) and (10–11) pole figures obtained by neutron diffraction: (**a**) AZ31; (**b**) AZ81; and (**c**) AZ91 sheets.

The bulk textures of the as-extruded AZ31, AZ81 and AZ91 alloy sheets were measured by neutron diffraction and further analyzed mainly using (10–10), (0002) and (10–11) pole figures, as shown in Figure 7. It can be seen from Figure 7 that a very strong and uneven texture is formed in the AZ31 alloy, and the texture intensity at the edge area is much higher than that at the 1/4 and center areas. Nevertheless, the texture in the AZ81 and AZ91 sheets is obviously weakened, meanwhile the texture intensity of the AZ91 alloy is slightly higher than the AZ81 alloy, which is consistent with the EBSD results. According to the inverse pole figure in Figure 6, the AZ31 alloy sheet contains a large number of coarse grains, indicating that the recrystallized grains grow up during hot extrusion. It means the volume fraction of some preferentially oriented grains increases and the texture is enhanced. Both AZ81 and AZ91 alloy sheets possess a finer and more homogeneous microstructure compared to AZ31 due to the increase in the Al content in the alloy. Usually, the fine recrystallized grains have more random orientation, which is the reason that the texture intensity of the AZ81 and AZ91 alloy sheets is reduced. Moreover, the distribution of basal pole is more homogeneous for AZ81 and AZ91 in different areas, which may be related to their relatively uniform microstructure.

**Figure 8:**
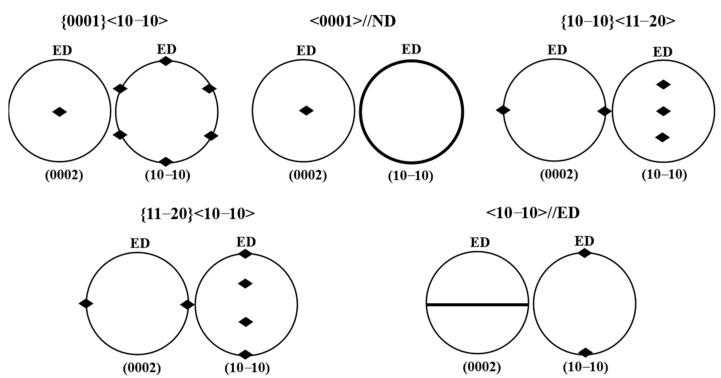
Five typical texture components exist in rectangular extrusion Mg alloy sheet.

Usually, rectangular extrusion is a deformation mode, which combines texture components of rolling and round extrusion following orthorhombic sample geometry [40]. Figure 8 gives several typical texture components that may occur in rectangular extrusion Mg alloy sheets. If a combination of both types is generated, the influence on the material properties depends on the texture sharpness of both texture types [41].

From Figure 7a, one can see that the four texture components shown in Figure 8 exist at the 1/4 and center areas of the AZ31 alloy sheet. Firstly, the {0001} <10–10> is seen in the pole figure center of the basal plane (0002). The equivalent is the distribution of six poles in the (10–10) pole figure at the outer ring. The second component is the {11–20} <10–10> with four poles distributed along the ED over the ND to -ED in the (10–10) plane. The third component is the {10–10} <11–20> characterized by three poles distributed alone the line of ED over the ND to -ED in the (10–10) plane, and accordingly, with two poles located at the outer ring along the TD in the basal plane. The last weak texture component, namely the <10–10> fiber parallel to the ED, and for this texture component, there is an orientation girdle from the TD over the ND to -TD present in the basal plane. It is reduced to a partial girdle in the basal plane at the 1/4 area of the AZ31 alloy sheet. It should be noted that the texture distribution is more concentrated and only the {10–10} <11–20> texture component can be evidently founded at the edge area of the AZ31 alloy sheet. Figure 7b, c shows that there are four main texture components in the AZ81 and AZ91 alloy sheets. They are the {0001} <10–10>, {11–20} <10–10> and weak <0001>//ND component. Meanwhile, the weak texture component of <10–10>//ED is also formed, but it is even not directly visible in the basal plane at the 1/4 and center areas in the AZ91 alloy sheet. The central pole in the basal plane (0002) is strongest coming from an overlap of {0001} <10–10> with a <0001> fiber texture parallel to ND. The <10–10> fiber texture component parallel ED is reduced to a partial girdle in the (0002) plane. The mixture of both <0001> fiber//ND and {0001} <hkil> ideal components are common and reflect the deformation part and the recrystallization part of the texture during high temperature extrusion [42]. There is no texture component of the <0001> fiber//ND in AZ31, proving that complete recrystallization occurs in the AZ31 alloy sheet.

It is generally accepted that the critical resolved shear stresses (CRSSs) of the basal 〈a〉 slip mode is much lower than those of the non-basal slips in hcp Mg at room temperature. Therefore, the low-temperature plastic deformation of Mg alloys occurs principally via the basal slip, which is also responsible for the formation of a strong basal texture or near basal texture in deformed Mg alloys. However, it was also reported that the CRSS of non-basal slips decreases substantially with an increase in temperature. In this study, AZ31, AZ81 and AZ91 alloys were extruded at a high temperature (~420–430 °C for AZ31 and ~360–370 °C for AZ81 and AZ91), therefore, besides the basal slip, the non-basal slips, such as prismatic slip <c+a>, should be considered as a mechanism contributing to deformation behavior. As a result, the production of a strong basal texture and other components of texture in the three as-extruded Mg alloys is attributed to the coordination and completion effects of basal and non-basal slips. It is believed that the DRX also contributes to texture development.

Normally, even if it has similar or nearly identical processing of rectangular extrusion, textures develop very differently for different alloys. From a crystallographic point of view, alloying elements can go as solutes in the crystal structure of Mg, influencing the generation of point, linear and planar defects and having a heavy impact on the deformation and recrystallization behavior. Moreover, alloying elements could be involved in the formation of second phase particles, having a great influence on deformation and recrystallization. In this study, Al atoms mainly exist in AZ31 alloy sheets in the form of solutes, while in AZ81 and AZ91, they may exist in the form of precipitates and solutes. This might be one part of the explanation of the texture variation observed in AZ31, AZ81 and AZ91.

It is noteworthy that the texture distribution in the AZ81 and AZ91 sheets obtained by EBSD is exactly the same as that obtained by neutron diffraction. However, for the AZ31 sheet, the (0002) pole figures achieved by the two techniques are not quite the same. Figure 6a shows that there are plenty of coarse grains in the AZ31 sheet; therefore, a relatively small number of grains are involved in texture measurement by EBSD, which is usually used to observe micro-regions or sheet surfaces, showing a lack of representativeness in texture characterization, leading to the statistics being relatively poor in this study. The highest quality of measured textures can be obtained by neutron diffraction due to the excellent grains statistics and the large test volume [38,43]. It is particularly beneficial to measure the texture of materials containing coarse grains. So, the pole figures measured by neutrons in our study can better represent the texture information in Mg alloy sheets. Both results from EBSD and neutron diffraction show that the texture intensity of AZ81 is lower in comparison to AZ91, and a very uneven texture is formed in the AZ31 alloy sheet.

### 3.3. Mechanical Properties

**Figure 9:**
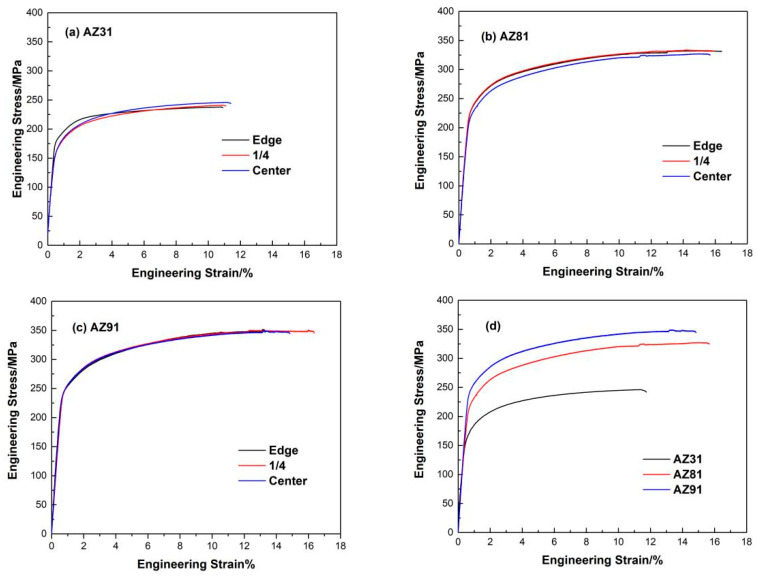
Stress–strain curves of as-extruded three Mg alloy sheets in different positions: (**a**) AZ31 alloy, (**b**) AZ81 alloy, (**c**) AZ91 alloy and (**d**) center area of three alloy sheets.

Engineering tensile stress–strain curves of three as-extruded Mg alloy sheets in different areas at room temperature are presented in Figure 9. It can be seen that relatively high values of YS and UTS are achieved in AZ81 and AZ91 alloy sheets through hot extrusion, and the mechanical properties are relatively uniform at different areas. Table 3 gives the recorded tensile properties of three as-extruded Mg alloy sheets. The YS and UTS of AZ81 and AZ91 alloy sheets are all enhanced compared with AZ31 alloy sheets, especially the UTS of AZ81 and AZ91 which is much higher than AZ31. Note that although AZ91 has the highest YS and UTS, the UE at different areas varies relatively greatly. In general, the AZ81 alloy sheet, which has a UTS of about 330MPa and UE of about 16% at room temperature, exhibits the most excellent comprehensive mechanical properties of all three alloy sheets.

Usually, the strengthening mechanisms of materials include grain boundary strengthening, second phase strengthening, solid solution strengthening and so on. In this study, grain refinement should be an effective strengthening method for AZ81 and AZ91 alloy sheets due to the finer and finer grains with the increasing Al content. According to the Hall–Petch relation: Δσ_grain_ = kd^−1/2^, where d is the average grain size and k is the stress concentration factor [44,45,46]. The strength contribution from grain boundaries on YS for AZ81 and AZ91 are much larger than it for AZ31 due to the much finer grain size of AZ81 and AZ91. It indicates that the addition of Al leads to a major increase in grain boundary strengthening. Moreover, the interaction between numerous fine precipitates of Mg_17_Al_12_ particles and locations in AZ81 and AZ91 sheets can hinder the movements of dislocations, which improves the deformability and thus enhances YS by the Orowan mechanism, while the coarse precipitates of Al_2_Mn in AZ31 are not beneficial for the tensile properties’ enhancement. In conclusion, both fine α-Mg grains and the dispersion of Mg_17_Al_12_ precipitates are responsible for the high YS of AZ81 and AZ91 alloy sheets [47].

From Table 3 one can see that the UTS of AZ81 and AZ91 sheets increases by more than 100 MPa compared with AZ31 sheets. It has been reported that a high volume fraction of well-dispersed fine Mg_17_Al_12_ particles in the Mg alloy could remarkably retard the recovery of dislocations, having a great influence on the increase of the dislocation density and the associated evolution of dislocation substructures. Therefore, forcing a dislocation to interact with them and increase the corresponding dislocation storage capability [9] is known to enhance the UTS of metallic materials. Thus, the presence of a large number of fine Mg_17_Al_12_ particles in AZ81 and AZ91 alloy sheets is beneficial to strong work hardening, which promotes both high UTS and ductility.

Table 3 shows that the UE of an AZ31 sheet is about 11% and it is about 16% for an AZ81 sheet. So, the UE has an obvious improvement for AZ81 compared with AZ31. For the AZ91 sheet, the UE at the edge, 1/4 and center area is about 13%, 16% and 15%, respectively. Even though the UE of AZ91 is inhomogeneous in different areas, it is still higher than that of AZ31. The previous study showed that refined grains and precipitates are beneficial for improving the fracture elongation of Mg alloys. As shown in Figure 6, the refined microstructure is formed in the AZ81 and AZ91 sheets; moreover, numerous Mg_17_Al_12_ particles are precipitated in two alloy sheets, implying that refined grains and precipitates are one of the main reasons for the enhanced elongation of the AZ81 and AZ91 sheets. On the other hand, the texture is another main factor that can affect mechanical properties and it has been proved that weak basal texture is conducive to improving the ductility of Mg alloy sheets [3]. Figure 7 reveals that the AZ81 alloy sheet exhibits the weakest basal texture, followed by the AZ91 alloy sheet, and the AZ31 alloy sheet presents the strongest basal texture among all three sheets. It is the reason why the AZ91 alloy sheet, having the smallest average grain size, shows moderate elongation. It can be concluded that the comprehensive factors such as basal texture, microstructure and precipitates determine the UE of the three Mg alloy sheets.

As mentioned earlier, the anisotropy of Mg alloys is strongly affected by the deformation texture, which varies in different deformation routes (e.g., extrusion, rolling, forging) [48]. Therefore, it is important to reveal the texture and the anisotropy that develop at different deformation routes for optimized texture characteristics and improved mechanical properties. Xu et al. [23] studied the texture and mechanical properties of the AZ91 alloy prepared by hot extrusion with equal channel angular pressing. They found that the extrusion AZ91 alloy had a YS of 244 MPa, UTS of 357.3 MPa and UE of 11.6% along the ED, while along the TD and ND, the YS was 122MPa, UTS was 154 MPa and UE was 2.7%, which exhibits a significant strong anisotropy. They thought the anisotropic mechanical behavior could be explained by its texture. Wang et al. [49] investigated the microstructures, textures and mechanical properties of AZ31 Mg alloy sheets fabricated by asymmetric porthole die extrusion dies with different titled angles. The AZ31 fabricated by the die entitled 90° had a YS of 163.3 MPa, UTS of 350.1 MPa and UE of 22.8% in the tensile directions of 0°, a YS of 178.4 MPa, UTS of 394.6 MPa and UE of 27.1% in the tensile directions of 45° and a YS of 206.9 MPa, UTS of 357.9 MPa and UE of 24.3% in the tensile directions of 90°. The results showed that the weak tilted basal texture caused the lower yield strength, but the higher elongation to failure was attributed to the combination of grain refinement and texture weakening. Zhao et al. [32] studied the texture weakening mechanism and mechanical properties of the AZ80 Mg alloy processed by repetitive upsetting-extrusion. After secondary extrusion, they got the extruded AZ80 with a YS of 192 MPa, UTS of 294 MPa and UE of 14.3% along the ED, and a YS of 186 MPa, UTS of 289 MPa and UE of 9.5% along the TD. They believed that the weakened texture reduced the TYS anisotropy of the extruded rods between loading along the ED and the TD.

The study in the above literature showed that texture obviously affected the anisotropy of tensile mechanical properties for Mg alloys in different direction, and this effect can be effectively reduced by weakening the basal texture. In this work, the tensile mechanical properties were tested in ED for three Mg alloys. In the next step of the work, the tensile mechanical properties will be tested in the TD to further investigate the texture effect on the anisotropy of Mg alloy sheets, which is helpful to make further improvements on mechanical properties.

## 4. Conclusions

In the present work, wide Mg alloy sheets with different Al contents of AZ31, AZ81 and AZ91 were fabricated by hot extrusion. High UTS and EU were simultaneously obtained in AZ81 and AZ91 alloy sheets. The effect of the Al content on the microstructure, bulk texture and tensile properties of three as-extruded wide Mg-Al-Zn alloy sheets were investigated. The conclusions can be summarized as follows:

(1) The relatively large average grain size of the extruded AZ31 alloy sheet is mainly due to the lack of pinning effect from dispersed Mg_17_Al_12_ precipitates on grain growth and the higher extrusion temperature applied. With the increase in the Al content, the grain size is greatly refined, which attributes to Mg_17_Al_12_ particles at the grain boundaries effectively hindering the growth of DRXed α-Mg grains in AZ81 and AZ91 alloy sheets.
(2) The AZ31 alloy sheet exhibits a strong and uneven texture in the basal plane, while the texture distribution is more homogenous and texture intensity is significantly weakened in the AZ81 and AZ91 alloy sheets due to the homogeneous microstructure and finer recrystallized grains having a more random orientation with the increase in the Al contents.
(3) With the increasing Al content, a high UTS of ~327–352 MPa and large elongation of ~13.3–16.4% for as-extruded AZ81 and AZ91 alloy sheets are mainly ascribed to combination factors of the refined microstructure, Mg_17_Al_12_ precipitates and a weakened basal texture.

## Figures and Tables

**Table 1:** Typical penetration depth and sample volume of neutrons, X-rays and Electrons.

	Neutrons	X-rays	Electrons
Penetration depth (mm)	10	10^−1^–10^−2^	10^−4^
Sample volume (mm^3^)	10^3^	10^−1^–10^−6^	10^−10^–10^−16^

**Table 2:** The compositions of studied alloys.

Normal Composition	Measured Composition (wt.%)			
	Al	Zn	Mn	Mg
AZ31	2.91	0.54	0.25	Bal.
AZ81	7.78	0.51	0.28	Bal.
AZ91	8.73	0.56	0.23	Bal.

**Table 3:** Mechanical properties of three different Mg alloys.

Sample	YS (MPa)			UTS (MPa)			UE (%)		
	Center	1/4	Edge	Center	1/4	Edge	Center	1/4	Edge
AZ31	$227_{-2}^{+3}$	$223_{-2}^{+2}$	$224_{-4}^{+3}$	$246_{-1}^{+2}$	$241_{-2}^{+4}$	$238_{-3}^{+1}$	$11.4_{-0.5}^{+1.6}$	$11.1_{-2.8}^{+2.1}$	$11.0_{-1.6}^{+1.5}$
AZ81	$265_{-5}^{+6}$	$268_{-8}^{+5}$	$275_{-10}^{+7}$	$327_{-1}^{+3}$	$334_{-1}^{+1}$	$332_{-3}^{+1}$	$15.7_{-0.4}^{+1.8}$	$15.9_{-2.9}^{+2.2}$	$16.4_{-2.1}^{+1.2}$
AZ91	$268_{-1}^{+1}$	$282_{-3}^{+3}$	$278_{-3}^{+1}$	$349_{-1}^{+1}$	$351_{-1}^{+1}$	$352_{-2}^{+2}$	$14.8_{-1.2}^{+1.5}$	$16.4_{-0.7}^{+0.8}$	$13.3_{-0.4}^{+1.8}$
